# Prevalence of Severe Maternal Morbidity and Factors Associated With Maternal Mortality in Ontario, Canada

**DOI:** 10.1001/jamanetworkopen.2018.4571

**Published:** 2018-11-09

**Authors:** Joel G. Ray, Alison L. Park, Susie Dzakpasu, Natalie Dayan, Paromita Deb-Rinker, Wei Luo, K. S. Joseph

**Affiliations:** 1 Department of Medicine, St. Michael’s Hospital, University of Toronto, Toronto, Ontario, Canada; 2 Department of Obstetrics and Gynaecology, St. Michael’s Hospital, University of Toronto, Toronto, Ontario, Canada; 3 Institute for Clinical Evaluative Sciences, Toronto, Ontario, Canada; 4 Maternal, Child and Youth Health Unit, Public Health Agency of Canada, Ottawa, Ontario, Canada; 5 Department of Medicine, McGill University, Montreal, Quebec, Canada; 6 Department of Epidemiology, McGill University, Montreal, Quebec, Canada; 7 Research Institute, McGill University Health Centre, Montreal, Quebec, Canada; 8 Department of Obstetrics and Gynaecology, School of Population and Public Health, University of British Columbia, Vancouver, British Columbia, Canada

## Abstract

**Importance:**

Severe maternal morbidity is defined by potentially life-threatening conditions. The association between the number of severe maternal morbidity (SMM) indicators and maternal death is not known.

**Objective:**

To quantify the association between the number of SMM indicators and maternal mortality.

**Design, Setting, and Participants:**

This population-based cohort study used provincial databases for data on all live birth and stillbirth hospital deliveries among women in Ontario, Canada, from April 1, 2002, to February 18, 2017. Excluded from this cohort were those with invalid identification number, non–Ontario residency, maternal age younger than 10 years or older than 55 years or unknown, or gestational age fewer than 20 weeks or unknown as well as any out-of-hospital births, ectopic pregnancies, or spontaneous or induced abortions.

**Exposures:**

Number of SMM indicators identified between 20 weeks’ gestation and 42 days after the index delivery.

**Main Outcomes and Measures:**

Maternal death occurring from delivery to 42 days after the index delivery.

**Results:**

Of the 1 953 943 total births among 1 211 396 women, 181 maternal deaths occurred within 42 days after birth, a rate of 9.3 per 100 000 births. Of the 181 women who died, 123 (68.0%) had at least 1 SMM indicator compared with 1.7% (33 152) of women who survived. Standardized differences suggested that women who died, compared with the women who lived, were older (mean [SD] age, 31.0 [6.2] years vs 30.1 [5.5] years; standardized difference, 0.15) and more likely to reside in a lower-income area (99 [54.7%] vs 832 231 [42.6%]; standardized difference, 0.24), be nulliparous (93 [51.4%] vs 880 386 [45.1%]; standardized difference, 0.13), and be of Afro-Caribbean origin (12 [6.6%] vs 64 948 [3.3%]; standardized difference, 0.15). The most frequent SMM indicators were intensive care unit admission (81 [44.8%]), invasive ventilation (77 [42.5%]), cardiac conditions (69 [38.1%]), complications of obstetric surgery or procedures (32 [17.7%]), and postpartum hemorrhage with blood transfusion (31 [17.1%]). The rate of maternal mortality increased exponentially with the number of SMM indicators: 0 indicators (3.0 per 100 000 births), 1 (71.7 per 100 000 births), 2 (385.9 per 100 000 births), 3 (1274.2 per 100 000 births), 4 (2236.8 per 100 000 births), 5 (4285.7 per 100 000 births), and 6 or more (9422.5 per 100 000 births). Adjusted relative risks for maternal death ranged from 20.1 (95% CI, 11.6-34.7) with 1 SMM indicator to 2192.0 (95% CI, 1287.0-3735.0) with 6 or more SMM indicators compared with 0 indicators.

**Conclusions and Relevance:**

Maternal death may be associated with the number of SMM indicators and occur in certain identifiable groups of women; targeting preventable SMM indicators or limiting their progression may reduce the number of maternal deaths.

## Key Points

**Question:**

What is the association between the number of indicators of severe maternal morbidities and maternal mortality in Ontario, Canada?

**Findings:**

In a population-based cohort study of 1.9 million hospital births in Ontario, Canada, the number of severe maternal morbidity indicators was associated with all-cause mortality within 42 days after the index delivery. Adjusted mortality was higher among women with 1 severe maternal morbidity indicator, those with 2 indicators, and those with 6 or more indicators compared those with 0 indicators.

**Meaning:**

The number of severe maternal morbidity indicators may be associated with the risk of maternal death.

## Teaser

This cohort study evaluates 15 years’ worth of hospital obstetric delivery records from national health databases in Ontario, Canada, to examine the association of severe maternal morbidity indicators with maternal mortality among women shortly after childbirth.

## Introduction

Increasing attention has been focused on maternal mortality. The Millennium Development Goal, which aimed to reduce maternal deaths by 75% between 1990 and 2015, led to substantial progress in many countries, primarily through improved family planning and obstetric care.^1^ Between 1990 and 2013, the maternal mortality ratio declined in some industrialized countries^1^ but remained stable in Canada (8 deaths per 100 000 births) and increased in the United States (18 deaths per 100 000 births).^2,3^ As many as half of maternal deaths in high-income countries are thought to be preventable, especially deaths associated with hemorrhage and complications due to preexisting chronic conditions.^4^

Severe maternal morbidity (SMM) is identified by conditions that are along the continuum to maternal death,^5^ including life-threatening and disabling diseases, organ dysfunction, and/or receipt of invasive therapy, during pregnancy or within 42 days after delivery.^6^ Similar to maternal death, SMM is increasing.^7^ Between 2003 and 2010, the rate of SMM was approximately 13 to 15 per 1000 births in Canada^8^ and 11 to 16 per 1000 births in the United States.^9^

A strong argument can be made in favor of conducting SMM surveillance as an adjunct to maternal mortality surveillance: SMM is more prevalent than death,^8^ and some SMM components, including cerebrovascular disease, acute hemodialysis, obstetric embolism, and assisted ventilation, are associated with death.^10^ Targeting preventable SMM or limiting its progression once realized could lead to a reduction in maternal deaths.^8,11^ A better understanding of the association between SMM and maternal deaths is needed, which was the purpose of the current study.

## Methods

### Study Design

This population-based cohort study used hospital obstetric delivery records identified within administrative health data sets,^11^ which are housed at the Institute for Clinical Evaluative Sciences and described in the eTable in the Supplement. All data sets were linked using unique encoded identifiers and analyzed at the Institute for Clinical Evaluative Sciences. Use of data for this study was authorized under section 45 of Ontario’s Personal Health Information Protection Act, which does not require review by a research ethics board or patient informed consent. This study followed the Strengthening the Reporting of Observational Studies in Epidemiology (STROBE) reporting guideline.

### Participants

There were 1 965 529 live birth or stillbirth hospital deliveries between April 1, 2002, and February 18, 2017, in the province of Ontario, Canada, where the Ontario Health Insurance Plan (OHIP) includes universal coverage of prenatal and obstetric care for all Ontario residents. Included in this study were 1 953 943 individual deliveries among 1 211 396 women, and 11 586 births (0.6%) were excluded because of an invalid maternal OHIP number or hospitalization number, non–Ontario residency, maternal age younger than 10 years or older than 55 years or unknown, or gestational age fewer than 20 weeks or unknown, each at the time of the index delivery (eFigure in the Supplement). Also excluded were any out-of-hospital births, ectopic pregnancies, or spontaneous or induced abortions before 20 weeks’ gestation because they might not be as completely documented in the current administrative databases.

The Canadian Institute for Health Information Discharge Abstract Database and the National Ambulatory Care Reporting System database were used to capture all hospital admissions and emergency department visits, including obstetric deliveries, with up to 25 diagnostic and procedural codes arising within a hospitalization; preexisting health conditions up to 1 year before the delivery; and maternal demographic characteristics (eTable in the Supplement). The labor and delivery data in the Discharge Abstract Database have been validated^10^ and used in many previous studies.^3,8,11^ Diagnostic codes are based on the *International Statistical Classification of Diseases and Related Health Problems, Tenth Revision, Canada* (*ICD-10-CA*), and procedural codes are based on the *Canadian Classification of Health Interventions*. Preexisting health conditions were also captured in the OHIP Database, which contains *International Classification of Diseases, Ninth Revision* (*ICD-9*) diagnostic codes for all outpatient visits. Deaths occurring between April 1, 2002, and March 31, 2017, were identified from the Office of the Registrar General Vital Statistics-Death registry and from the Ministry of Health Registered Persons Database, which contains vital status and sociodemographic information for all individuals ever eligible for OHIP. The Immigration, Refugees and Citizenship Canada Permanent Resident Database was used to identify maternal world region of origin as a proxy for ethnicity. This database contains data on country of citizenship for immigrants to Canada from 1985 onward. Women not linked to this database were classified as Canadian born. Income quintile and rural residence data were from the Statistics Canada census.

### Exposures and Outcomes

The main exposure was the number of SMM indicators identified in a given pregnancy, between 20 weeks’ gestation and 42 days after the index delivery. We adapted a definition of SMM that was previously developed by the Canadian Perinatal Surveillance System,^8^ which includes 44 unique indicators based on *ICD-10-CA* and *Canadian Classification of Health Interventions* procedural codes (eg, intensive care unit [ICU] admission, eclampsia, and use of assisted ventilation) and that is similar to the approach developed at the US Centers for Disease Control and Prevention.^12^ After 4 indicators of death were removed from the main exposure, the maximum number of SMM indicators was 40. The study outcome was all-cause maternal mortality at the index birth or up to 42 days thereafter.

### Statistical Analysis

Means (SDs) and proportions of baseline variables were compared between women who died and women who were alive within 42 days after the index delivery, with a standardized difference greater than 0.10 indicating an important difference. We also calculated the median (interquartile range [IQR]) number of SMM indicators and the prevalence of any SMM, and we ranked the top 10 most frequent SMM indicators among women who died and those who lived.

The maternal mortality rate was calculated per 100 000 total births (comprising live births and stillbirths) among women with 0 (referent), 1, 2, 3, 4, 5, or 6 or more SMM indicators during the index pregnancy. Corresponding relative risks (RR) and 95% CIs were estimated using modified Poisson regression with a robust error variance. Generalized estimating equations with an exchangeable correlation structure accounted for correlated errors in the case of multiple pregnancies for the same woman. Relative risks were adjusted for the following, each at the time of the index delivery: maternal age (15-19, 20-29, 30-39, or 40-55 years), neighborhood income quintile (Q1-Q2 or unknown vs Q3-Q5), rural residence (rural or unknown vs urban), stillbirth, multifetal pregnancy, and world region of origin (Caribbean/Africa, South Asia, East Asia, other, or Canadian born) as well as maternal diabetes, chronic hypertension, renal disease, and illicit drug or tobacco use within 365 days before the index delivery. The proportion of missing data was 0.5% for income quintile, 0.01% for rural residence, and less than 0.01% for parity. After removal of missing covariates, we observed no meaningful association with death rates or RR estimates.

All analyses were run using SAS statistical software, version 9.4 for UNIX (SAS Institute Inc).

## Results

In total, 181 maternal deaths occurred among all 1 953 943 births, corresponding to a rate of 9.3 per 100 000 births (Table 1). Standardized differences suggested that women who died within 42 days after delivery, compared with the women who lived, were older (mean [SD] age, 31.0 [6.2] years vs 30.1 [5.5] years; standardized difference, 0.15) and more likely to reside in a lower-income area (99 [54.7%] vs 832 231 [42.6%]; standardized difference, 0.24), be nulliparous (93 [51.4%] vs 880 386 [45.1%]; standardized difference, 0.13), and be of Afro-Caribbean origin (12 [6.6%] vs 64 948 [3.3%]; standardized difference, 0.15). In comparing women who died with those who lived, the rate of stillbirth was higher (31 [17.1%] vs 11 403 [0.6%]), as were rates of multifetal pregnancies (11 [6.1%] vs 35 890 [1.8%]), preeclampsia (14 [7.7%] vs 27 313 [1.4%]), gestational hypertension (17 [9.4%] vs 78 828 [4.0%]), illicit drug or tobacco use (7 [3.9%] vs 34 744 [1.8%]), preexisting diabetes (30 [16.6%] vs 141 349 [7.2%]), hypertension (23 [12.7%] vs 66 963 [3.4%]), and renal disease (15 [8.3%] vs 5390 [0.3%]) (Table 1).

**Table 1.** Characteristics and SMM Indicators Among Women Who Died or Lived Within 42 Days of the Index Delivery, 2002-2017^a^

Characteristic	Death ≤42 Days After Delivery (n = 181)	No Death ≤42 Days After Delivery (n = 1 953 762)	Standardized Difference
Age, mean (SD), y	31.0 (6.2)	30.1 (5.5)	0.15
Age category, y			
15-19	6 (3.3)	64 567 (3.3)	0
20-29	67 (37.0)	795 361 (40.7)	−0.08
30-39	93 (51.4)	1 020 504 (52.2)	−0.02
40-55	15 (8.3)	73 330 (3.8)	0.19
Low-income quintile 1-2 or unknown	99 (54.7)	832 231 (42.6)	0.24
Rural residence or unknown	23 (12.7)	202 112 (10.3)	0.07
World region of origin			
Caribbean/Africa	12 (6.6)	64 948 (3.3)	0.15
South Asia	11 (6.1)	149 222 (7.6)	−0.06
East Asia	14 (7.7)	113 254 (5.8)	0.08
Canadian born	129 (71.3)	1 459 640 (74.7)	−0.08
Other	15 (8.3)	166 698 (8.5)	−0.01
Parity, median (IQR)	0 (0-1)	1 (0-1)	−0.04
Nulliparity	93 (51.4)	880 386 (45.1)	0.13
Stillbirth	31 (17.1)	11 403 (0.6)	0.61
Multifetal pregnancy	11 (6.1)	35 890 (1.8)	0.22
Any preeclampsia	14 (7.7)	27 313 (1.4)	0.31
Gestational hypertension	17 (9.4)	78 828 (4.0)	0.22
Conditions in the year before the index delivery			
Diabetes	30 (16.6)	141 349 (7.2)	0.29
Chronic hypertension	23 (12.7)	66 963 (3.4)	0.35
Renal disease	15 (8.3)	5390 (0.3)	0.40
Illicit drug or tobacco use	7 (3.9)	34 744 (1.8)	0.13
SMM indicators			
Median (IQR)	2 (0-5)	0 (0)	1.36
≥1 SMM indicator	123 (68.0)	33 152 (1.7)	1.94

Abbreviations: IQR, interquartile range; SMM, severe maternal morbidity.

^a^ All data are presented as No. (%) unless otherwise indicated.

Severe maternal morbidity that arose during pregnancy or after delivery was more likely in women who died than in those who did not (123 [68.0%] vs 33 152 [1.7%]), with a median (IQR) number of SMM indicators of 2 (0-5) and 0 (0), respectively (Table 1). Postpartum hemorrhage with blood transfusion (45.3 per 10 000 births), ICU admission (33.0 per 10 000 births), puerperal sepsis (28.8 per 10 000 births), severe preeclampsia or HELLP (hemolysis, elevated liver enzyme levels, and low platelet count) syndrome (15.9 per 10 000 births), and hysterectomy (15.3 per 10 000 births) were the most common SMM indicators (Figure). The highest rate of maternal mortality was seen among those who had received dialysis (100 per 1000 births affected) or who experienced hepatic failure (86.0 per 1000 births affected), disseminated intravascular coagulation (82.8 per 1000 births affected), cerebrovascular disease (80.9 per 1000 births affected), or adult respiratory distress syndrome (54.9 per 1000 births affected) (Figure).

**Figure.**
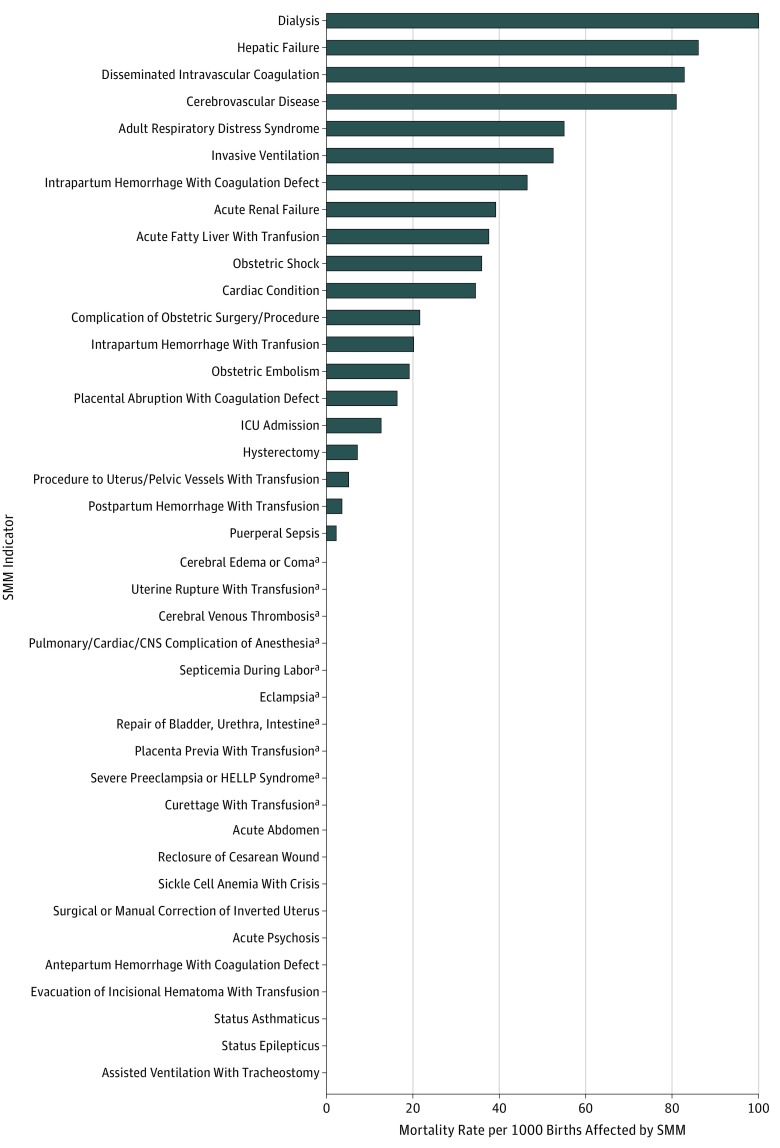
Rate of Maternal Mortality by Severe Maternal Morbidity (SMM) Indicator The SMM indicators are listed in order of decreasing mortality rate. CNS indicates central nervous system*;* HELLP, hemolysis, elevated liver enzyme levels, and low platelet count; and ICU, intensive care unit. ^a^Indicators with 1 to 5 deaths were suppressed variables.

Among women who died, the most frequent SMM indicators were ICU admission (81 [44.8%]), invasive ventilation (77 [42.5%]), cardiac conditions (69 [38.1%]), complications of obstetric surgery or procedures (32 [17.7%]), and postpartum hemorrhage with blood transfusion (31 [17.1%]) (Table 2). Some of these same SMM indicators were also seen in women who lived, albeit at lower rates; however, acute renal failure (29 [16.0%]), cerebrovascular disease (25 [13.8%]), obstetric shock (20 [11.0%]), and obstetric embolism (15 [8.3%]) were seen in the top 10 list of indicators only among women who died but not among those who lived (Table 2).

**Table 2.** Top 10 Most Common SMM Indicators, Ranked According to Their Prevalence Among Women Who Died or Lived Within 42 Days of the Index Delivery, 2002-2017^a^

Indicator Rank No.	SMM Indicator	No. (%)
**Death ≤42 Days After Delivery (n = 181)**		
1	ICU admission	81 (44.8)
2	Invasive ventilation	77 (42.5)
3	Cardiac condition	69 (38.1)
4	Complications of obstetric surgery or procedure	32 (17.7)
5	Postpartum hemorrhage with RBC transfusion	31 (17.1)
6	Acute renal failure	29 (16.0)
7	Cerebrovascular disease	25 (13.8)
8	Hysterectomy	21 (11.6)
9	Obstetric shock	20 (11.0)
10	Obstetric embolism	15 (8.3)
**No Death ≤42 Days After Delivery (n = 1 953 762)**		
1	Postpartum hemorrhage with RBC transfusion	8825 (0.5)
2	ICU admission	6376 (0.3)
3	Puerperal sepsis	5613 (0.3)
4	Severe preeclampsia or HELLP syndrome	3104 (0.2)
5	Hysterectomy	2961 (0.2)
6	Curettage with RBC transfusion	2170 (0.1)
7	Eclampsia	2018 (0.1)
8	Cardiac condition	1937 (0.1)
9	Complications of obstetric surgery or procedure	1457 (0.1)
10	Invasive ventilation	1392 (0.1)

Abbreviations: HELLP, hemolysis, elevated liver enzyme levels, and low platelet count; ICU, intensive care unit; RBC, red blood cell; SMM, severe maternal morbidity.

^a^ A birth may have had more than 1 SMM indicator.

The rate of maternal death per 100 000 births increased with the number of SMM indicators: 0 (3.0 per 100 000 births), 1 (71.7 per 100 000), 2 (385.9 per 100 000), 3 (1274.2 per 100 000), 4 (2236.8 per 100 000), 5 (4285.7 per 100 000), and 6 or more (9422.5 per 100 000) (Table 3). Unadjusted RRs ranged from 20.1 (95% CI, 11.6-34.7) with 1 SMM indicator to 2192.0 (95% CI, 1287.0-3735.0) with 6 or more SMM indicators compared with 0 SMM indicators. The corresponding adjusted RRs were only somewhat attenuated (Table 3).

**Table 3.** Association of Maternal Death Within 42 Days After Index Delivery With Number of SMM Indicators From 20 Weeks’ Gestation to 42 Days After Index Birth Hospitalization, 2002-2017

SMM Indicators, No.	Births Affected, No.	Deaths, No. (Rate per 100 000 Births)	Relative Risk of a Maternal Death (95% CI)	
			Unadjusted	Adjusted^a^
0	1 920 668	58 (3.0)	1.0 [Reference]	1.0 [Reference]
1	25 108	18 (71.7)	23.7 (14.0-40.3)	20.1 (11.6-34.7)
2	4923	19 (385.9)	127.8 (76.2-214.4)	101.6 (58.2-177.6)
3	1805	23 (1274.2)	422.0 (260.9-682.4)	323.3 (188.6-554.5)
4	760	17 (2236.8)	740.7 (433.4-1266.0)	585.2 (327.7-1045.0)
5	350	15 (4285.7)	1419.0 (812.3-2480.0)	1073.0 (582.2-1977.0)
≥6	329	31 (9422.5)	3120.0 (2045.0-4761.0)	2192.0 (1287.0-3735.0)

Abbreviation: SMM, severe maternal morbidity.

^a^ Adjusted for maternal age category (15-19, 20-29, 30-39, or 40-55 years), low-income area of residence (quintiles 1-2 or unknown), rural residence or unknown, stillbirth, multifetal birth, and world region of origin (Caribbean/Africa, South Asia, East Asia, Other, or Canadian born), each identified at the time of the index birth, as well as maternal diabetes, chronic hypertension, renal disease, and tobacco or illicit drug use within 365 days preceding the index birth.

## Discussion

In this large population-based study of women who had a hospital delivery within a universal health care setting, the rate of maternal death within 42 days after delivery was associated with the number of SMM indicators. The ranking of SMM indicators among those who died differed from that among those who lived, highlighting that certain procedures and conditions, such as invasive ventilation and cardiac disease, may be potentially more associated with fatalities than are others. Important demographic, preexisting condition, and pregnancy factors were also associated with maternal mortality.

### Other Studies

Our findings are consistent with findings from previous studies that identified characteristics associated with maternal death, including older age, African or Caribbean ethnicity,^13^ multifetal pregnancy,^14^ chronic medical conditions, and substance misuse.^13,15^ Stillbirths were more common among maternal deaths, consistent with other data,^15^ which underscores the need to include stillbirths and not just live births^1,2^ when calculating the risk of maternal death after delivery.

Similar to other research,^5,8^ this study found that women who died were more likely than those who lived to exhibit certain SMM indicators, including ICU admission, invasive ventilation, and cardiac conditions (Table 3). To our knowledge, no previous studies have examined the risk for mortality associated with the number of SMM indicators. Instead, previous studies explored the characteristics of women most likely to have an SMM indicator progress to death.^13^ For example, Kayem et al^13^ observed that women in the United Kingdom who had eclampsia, pulmonary embolism, amniotic fluid embolism, acute fatty liver of pregnancy, or antenatal stroke were more likely to die, especially those aged 35 years or older or who were of black race/ethnicity.

### Clinical and Policy Relevance

In Canada and the United States, the prevalence of SMM has increased in recent decades,^8,9^ as has the rate of maternal death, especially in the United States.^2^ Although deaths from amniotic fluid embolism might be more difficult to prevent, most deaths from hemorrhage, chronic conditions, and the hypertensive disorders of pregnancy should be preventable through improved preconception, prenatal, and intrapartum care.^4,16^ In US hospital systems with a comprehensive program designed to prevent maternal mortality, the rate of maternal death has been reduced to half the national average.^16^ Efforts have increasingly focused on developing maternal early warning systems and protocols to identify a woman’s clinical deterioration,^17^ alongside recommendations for managing or preventing worsening SMM and associated deaths.^2,8,18^ The Maternal Early Warning Trigger tool is an example of an early warning system that was found to substantially reduce SMM but not ICU admission.^12^ Adoption of concise diagnostic and treatment protocols for use in the labor and delivery and postpartum areas of a hospital, including rapid escalation of care, is a reasonable initiative for reducing SMM and maternal mortality.^12^ In addition, future studies may be needed to determine whether reducing the number of SMM indicators, both at a system level and an individual level, would be associated with a decrease in maternal mortality rate and/or hospital length of stay.

### Limitations and Strengths

The current study used validated data sets^10^ that captured more than 99% of all hospital births and accounted for all out-of-hospital deaths (in Ontario, Canada), which included a large number of maternal deaths over the 15-year study period. We lacked data on out-of-province deaths, as in the case of travel or emigration; however, it is uncommon for women to travel or emigrate within 42 days after delivery, heightening the probability that most deaths were accounted for in this study. Because the current study did not include pregnancies ending before 20 weeks’ gestation, such as an ectopic pregnancy or a spontaneous or induced abortion, we cannot comment on SMM or mortality for that subset of pregnant women. All-cause mortality was captured for births from 20 weeks onward, but complete data were lacking on the cause of death, including whether it was medical or accidental.

We observed a strong dose response in the risk of death with an increasing number of SMM indicators, but the 95% CIs were widened because of the relatively few number of fatalities (n = 58) among women with 0 SMM indicators, who formed the referent. Because *ICD-10-CA* coding specific to severe preeclampsia and the HELLP syndrome began in 2012, these indicators were undercaptured before 2012. Compared with *ICD-9*, however, the *ICD-10-CA* coding used here has improved the classification of severe preeclampsia and procedures to manage severe hemorrhage.

## Conclusions

Maternal death appears to be exponentially associated with the number of SMM indicators in pregnancy and after delivery. Targeting preventable SMM indicators, or limiting the progression of SMM once they are realized, may reduce maternal mortality.

## Notes

Supplement. **eTable.** Variables Used to Define Cohort Entry and Exclusion Criteria, Study Exposures, Outcomes and Adjustment Variables **eFigure.** Flow Chart of Cohort Inclusions and Exclusions
