# Supplementary material for: Prevalence of Severe Maternal Morbidity and Factors Associated With Maternal Mortality in Ontario, Canada
Source: JAMA Netw Open. 2018 Nov 9;1(7):e184571. doi: 10.1001/jamanetworkopen.2018.4571 (PMC6324398; doi:10.1001/jamanetworkopen.2018.4571)
Supplement: Supplement. — eTable. Variables Used to Define Cohort Entry and Exclusion Criteria, Study Exposures, Outcomes and Adjustment Variables eFigure. Flow Chart of Cohort Inclusions and Exclusions [file jamanetwopen-1-e184571-s001.pdf]

Supplementary Online Content

Ray JG, Park AL, Dzakpasu S, et al. Prevalence of severe maternal morbidity and factors associated with maternal mortality in Ontario, Canada. *JAMA Netw Open.* 2018;1(7):e184571. doi:10.1001/jamanetworkopen.2018.4571

eTable. Variables Used to Define Cohort Entry and Exclusion Criteria, Study Exposures, Outcomes and Adjustment Variables

eFigure. Flow Chart of Cohort Inclusions and Exclusions

This supplementary material has been provided by the authors to give readers additional information about their work.

©2018 Ray JG et al. *JAMA Network Open.*

eTable. Variables Used to Define Cohort Entry and Exclusion Criteria, Study Exposures, Outcomes and Adjustment Variables

Assessment	Timing	Disease or procedure or condition	ICD-9 [ICD-10-CA] codes in CIHI--DAD or NACRS[a]	OHIP ICD-9 diagnostic codes or fee codes {or other source if in parentheses}[a]	PubMed link to related validation studies for some codes
Cohort entry criterion	Index birth delivery hospitalization	Live birth or stillbirth obstetrical deliveries at ≥ 20 weeks' gestation, between April 1, 2002 and February 18, 2017	Main patient service code for "obstetrical delivery" in DAD (and linked to their newborn records in the ICES MOMBABY dataset: https://datadictionary.ices.on.ca/Applications/DataDictionary/Library.aspx?Library=MOMBABY)	--	--
Exclusion criteria	At the time of the index birth delivery hospitalization	Non-Ontario resident or invalid OHIP number	--	{Registered Persons Database (RPDB). See: https://datadictionary.ices.on.ca/Applications/DataDictionary/Library.aspx?Library=RPDB}	--
	Same	Maternal age < 10 or > 55 years, or missing	--	{RPDB}	--
Main study exposure	April 1, 2002 and March 31, 2017	Number of severe maternal morbidities between $20^{0/7}$ weeks' gestation and before 43 days after the index birth delivery	1. Severe preeclampsia and HELLP syndrome<br>2. Eclampsia<br>3. Cerebral venous thrombosis in pregnancy, or in the puerperium<br>4. Acute fatty liver with red blood cell (RBC) or plasma transfusion<br>5. Pulmonary, cardiac, and CNS complications of anaesthesia during	--	Previous definition by the Canadian Perinatal Surveillance System (https://www.ncbi.nlm.nih.gov/pmc/articles/PMC1216316 and

©2018 Ray JG et al. *JAMA Network Open.*

Assessment	Timing	Disease or procedure or condition	ICD-9 [ICD-10-CA] codes in CIHI--DAD or NACRS[a]	OHIP ICD-9 diagnostic codes or fee codes {or other source if in parentheses}[a]	PubMed link to related validation studies for some codes
			pregnancy, the puerperium, or labour and delivery<br>6. Placenta previa with hemorrhage with RBC transfusion<br>7. Placental abruption with coagulation defect<br>8. Antepartum hemorrhage with coagulation defect<br>9. Intrapartum hemorrhage with coagulation defect<br>10. Intrapartum hemorrhage with RBC transfusion<br>11. Rupture of the uterus with RBC transfusion, procedures to the uterus or hysterectomy<br>12. Postpartum hemorrhage with RBC transfusion, procedures to the uterus or hysterectomy<br>13. Cardiac conditions<br>14. Obstetric shock<br>15. Septicemia during labour<br>16. Complications of obstetric surgery and procedures<br>17. Puerperal sepsis<br>18. Obstetric embolism<br>19. Acute renal failure<br>20. Disseminated intravascular coagulation<br>21. Sickle cell anemia with crisis<br>22. Acute psychosis<br>23. Status epilepticus<br>24. Cerebral edema or coma<br>25. Cerebrovascular diseases: subarachnoid and intracranial hemorrhage, cerebral infarction, stroke<br>26. Status asthmaticus<br>27. Adult respiratory distress syndrome<br>28. Acute abdomen		https://www-ncbi-nlm-nih-gov/pubmed/21050516)

©2018 Ray JG et al. *JAMA Network Open.*

Assessment	Timing	Disease or procedure or condition	ICD-9 [ICD-10-CA] codes in CIHI--DAD or NACRS[a]	OHIP ICD-9 diagnostic codes or fee codes {or other source if in parentheses}[a]	PubMed link to related validation studies for some codes
			29. Hepatic failure<br>30. Assisted ventilation through endotracheal tube<br>31. Assisted ventilation through tracheostomy<br>32. Hysterectomy<br>33. Dialysis<br>34. Evacuation of incisional hematoma with RBC transfusion<br>35. Repair of bladder, urethra, or intestine<br>36. Procedures to the uterus/pelvic vessels with RBC transfusion<br>37. Surgical or manual correction of inverted uterus for vaginal births only<br>38. Reclosure of caesarean wound<br>39. Curettage with RBC transfusion<br>40. Maternal ICU admission		
Outcome	Date of death between 0 and 42 days after the index birth delivery date	All-cause maternal mortality	--	{RPDB} and {Office of the Registrar General Deaths (ORG-D). See: https://datadictionary.ices.on.ca/Applications/DataDictionary/Library.aspx?Library=ORGD }	--
Covariates	At the time of the index birth delivery hospitalization	Maternal age (years)	--	{RPDB}	--
	At the index birth delivery hospitalization	Maternal area-level income quintile	--	{Statistics Canada census data}	--
	Same	Maternal rural residence	--	{Statistics Canada census data}	--

©2018 Ray JG et al. *JAMA Network Open.*

Assessment	Timing	Disease or procedure or condition	ICD-9 [ICD-10-CA] codes in CIHI--DAD or NACRS[a]	OHIP ICD-9 diagnostic codes or fee codes {or other source if in parentheses}[a]	PubMed link to related validation studies for some codes
	Same	World region of origin:<br>1) Caribbean or Sub-Saharan Africa<br>2) South Asia<br>3) East Asia<br>4) Canada<br>5) Other	--	{Immigration, Refugees and Citizenship Canada (IRCC)'s Permanent Resident Database. See: https://datadictionary.ices.on.ca/Applications/DataDictionary/Library.aspx?Library=CIC}	--
	Same	Parity	CIHI-DAD (PREVBIRTH)	--	--
	Same	Multiple gestation	Z37.2-Z37.7, Z37.90, O30.0-O30.2, O30.8-O30.9, O31	--	--
	Same	Stillbirth	Z37.1, Z37.3, Z37.4, Z37.6, Z37.7, O36.4	--	--
	From 20 gestational weeks up to 42 days after the index birth delivery	Any preeclampsia	O11, O14	--	--
	Same	Gestational hypertension	O13	--	--
	Up to 1 year before the index birth delivery hospitalization	Diabetes mellitus	250, 648.8 [E10, E11, E13, E14, O244]	250	https://www.ncbi.nlm.nih.gov/pubmed/11874939
	Same	Chronic hypertension	401, 405, 642.0-642.2, 642.7 [I10, I15, O10, O11]	401	https://www.ncbi.nlm.nih.gov/pubmed/19858407

©2018 Ray JG et al. *JAMA Network Open.*

Assessment	Timing	Disease or procedure or condition	ICD-9 [ICD-10-CA] codes in CIHI--DAD or NACRS[a]	OHIP ICD-9 diagnostic codes or fee codes {or other source if in parentheses}[a]	PubMed link to related validation studies for some codes
	Same	Renal disease	584.5-584.9, 669.3, 958.5 634.3, 635.3, 636.3, 637.3, 638.3, 639.3, 250.4, 274.1, 403, 404, 405.01, 405.11, 405.91, 440.1, 446.21, 581, 582, 583, 585, 586, 587, 588.0, 588.8, 588.9, 590.0, 593.7, 791.0, 794.4 [N17, O08.4, T79.5, O90.4, E10.20, E10.21, E10.23, E11.20, E11.21, E11.23, M10.39, I12, I13, I15.0, I70.1, M31.0, N01, N03, N04, N05, N06, N07, N08, N11, N12, N13.7, N13.8, N13.9, N14, N15, N16, N18, N19, N25.0, N25.8, N25.9, N26, R80, R94.4]	403, 581, 585	https://www.ncbi.nlm.nih.gov/pubmed/23560464
	Same	Illicit drug or tobacco use	291, 292, 2940, 303, 304, 305, 648.3, 649.0, 6555, 980 [F10-F19, F55, G312, O354, O355, T51, T652, Z720, Z721, Z722]	291, 292, 303, 304, 305	

[a] CIHI Canadian Institute for Health Information; DAD Discharge Abstract Database; ICD-9 International Classification of Diseases, 9th Revision (before 2002/03); ICD-10-CA International Classification of Diseases, 10th Revision, Canada (2002/03 onward); ICU Intensive Care Unit; IRCC Immigration, Refugees and Citizenship Canada; NACRS National Ambulatory Care Reporting System; OHIP Ontario Health Insurance Plan; ORG-D Office of the Registrar General Deaths; RBC Red Blood Cell; RPDB Registered Persons Database

©2018 Ray JG et al. *JAMA Network Open.*

eFigure. Flow Chart of Cohort Inclusions and Exclusions

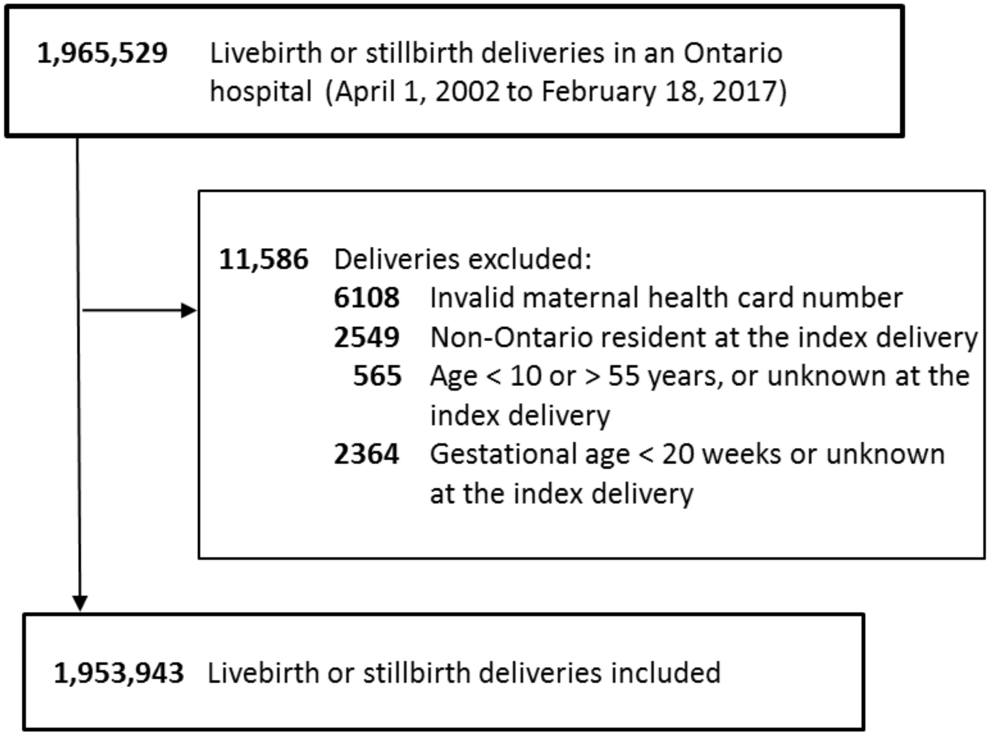

©2018 Ray JG et al. *JAMA Network Open.*
